# Inorganic Mercury Sequestration by a Poly(ethylene imine) Dendrimer in Aqueous Solution

**DOI:** 10.3390/molecules20033783

**Published:** 2015-02-26

**Authors:** Elena Salvador Serrano, Matteo Savastano, Antonio Bianchi

**Affiliations:** 1 Instituto de Ciencia Molecular, C/Catedrático José Beltrán 2, 46980 Paterna, Valencia, Spain; E-Mail: elena.salvador@uv.es; 2 Department of Chemistry “Ugo Schiff”, via della Lastruccia 3, 50019 Sesto Fiorentino, Italy; E-Mail: msavastano@unifi.it

**Keywords:** mercury, dendrimers, poly(ethylene imine), contamination, remediation

## Abstract

The interaction of the G-2 poly(ethylene imine) dendrimer L, derived from ammonia as initiating core, with Hg(II) and HgCl_4_^2−^ was studied in aqueous solution by means of potentiometric (pH-metric) measurements. Speciation of these complex systems showed that L is able to form a wide variety of complexes including 1:1, 2:1, 3:1 and 3:2 metal-to-ligand species, of different protonation states, as well as the anion complexes [(H_7_L)HgCl_4_]^5+^ and [(H_8_L)HgCl_4_]^6+^. The stability of the metal complexes is very high, making L an excellent sequestering agent for Hg(II), over a large pH range, and a promising ligand for the preparation of functionalized activated carbons to be employed in the remediation and the prevention of environmental problems.

## 1. Introduction

Mercury and most of its compounds are highly toxic. They can be absorbed through the skin and mucous membranes, while mercury vapours and volatile derivatives can be inhaled [1]. Mercury is mainly emitted into the air by industries that burn fossil fuels, particularly coal, and by incomplete incineration of wastes containing inorganic mercury. Over the years, however, different chemical manufacturers and other industrial facilities, have contributed to mercury contamination of the environment. A famous environmental disaster occurred in Japan, where dumping of mercury compounds into Minamata Bay from 1932 to 1968 caused severe poisoning symptoms or death to more than 3000 people from what became known as Minamata disease [2]. Concentrations of mercury up to 3.6 μg/L were measured in seawater of Minamata Bay from 1960 to 1962, when mercury was still being discharged into the bay [3,4], and this caused contamination levels as high as 35.7 ppm in marine products in the bay, while extremely higher mercury levels were found in the tissues (up to 705 ppm in hair) of the poisoned coastline inhabitants [5]. Notwithstanding that, one of the largest sources of mercury intake by people is still a fish-based diet, since fishes, in particular large size ones, accumulate mercury from the aqueous environments where they live.

Hg(II), its chloride complexes present in seawater and methylmercury(II), which is formed in the environment by microbial metabolism and by abiotic chemical reactions involving inorganic mercury, are the main polluting forms of mercury [6,7,8]. Considering the severe problems generated by this metal to biological systems, it is an important task to find methods of removing these undesirable compounds from the environment and from toxic waste and to find efficient chelation therapies to treat contaminated human beings.

**Figure 1:**
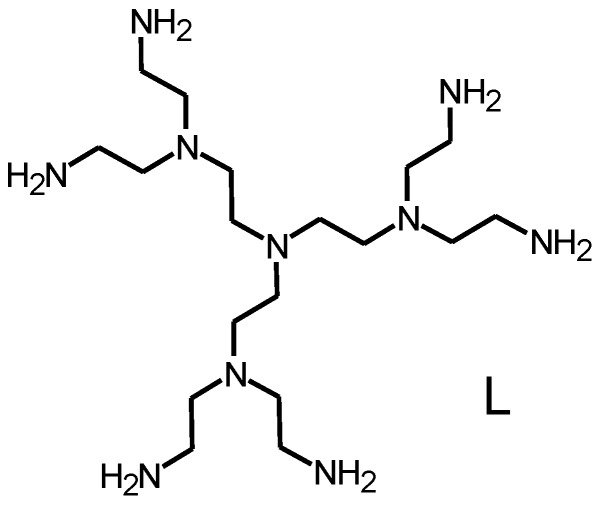
The G-2 poly(ethylene imine) dendrimer L.

It was shown that activated carbon functionalized with polyamine molecules can be efficiently used for the recovery of both metal ions [9,10,11] and inorganic anions [12], including HgCl_4_^2−^, from aqueous media. Such particular behaviour is due to the dual nature of the polyamine functionalities that are able to coordinate metal ions when they are completely or partially deprotonated and to bind anions, through electrostatic and hydrogen bonds, when they are extensively protonated. Among polyamines, dendrimeric ones are able to coordinate large numbers of metal ions, forming stable complexes, thanks to the many amine groups they contain and to their branched structures. For instance, we have recently shown that even the small poly(ethylene imine) dendrimer L (Figure 1) can bind two metal ions such as Ni(II), Zn(II) and Cd(II) and up to three Cu(II) ions [13]. The same ligand is also able to bind inorganic and organic anions and to form ion-pair complexes [14,15]. Accordingly, polyamine dendrimers display the appropriate binding properties for the recovery of both metal ions and anions from aqueous solutions and, as a matter of fact, it has been demonstrated that activated carbons functionalized with polyalkylamines display excellent performance as Pd(II) scavengers in water [10].

For this reason, we have studied the interaction of L with Hg(II) and HgCl_4_^2−^ to assess the ability of this ligand to sequestrate these ions in view of a possible use of the dendrimer to prepare functionalized activated carbons for the remediation of aqueous media contaminated by inorganic mercury. We describe here the results of this study.

## 2. Results and Discussion

Speciation of the Hg(II)/L systems and determination of the relevant stability constants were performed by means of pH-metric (potentiometric) titrations (0.1 M Me_4_NCl, 298.1 ± 0.1 K) and analysis of the associated data by means of the computer program HYPERQUAD [16] which furnished the stability constants collected in Table 1. A medium containing high chloride concentration (0.1 M) was adopted both to facilitate the determination of the large stability constants of Hg(II) complexes and to furnish information about Hg(II) complexation by L in aqueous media with high chloride levels, like seawater.

**Table 1:** Equilibrium constants (with standard deviations in parentheses) for the complexes formed by L with Hg(II) and HgCl_4_^2−^, determined in 0.1 M Me_4_NCl aqueous solution at 298.1 K.

Equilibria	log*K*
L + Hg^2+^ = HgL^2+^	28.17(5)
HgL^2+^ + H^+^ = HgLH^3+^	9.64(6)
HgLH^3+^ + H^+^ = HgLH_2_^4+^	8.91(7)
HgLH_2_^4+^ + H^+^ = HgLH_3_^5+^	8.45(5)
HgLH_3_^5+^ + H^+^ = HgLH_4_^6+^	6.13(4)
HgLH_4_^6+^ + H^+^ = HgLH_5_^7+^	4.85(5)
L + 2Hg^2+^ = Hg_2_L^4+^	48.38(8)
Hg_2_L^4+^ + H^+^ = Hg_2_LH^5+^	9.41(9)
Hg_2_LH^5+^ + H^+^ = Hg_2_LH_2_^6+^	6.63(6)
HgL^2+^ + Hg^2+^ = Hg_2_L^4+^	20.21(9)
2L + 3Hg^2+^ = Hg_3_L_2_^6+^	79.4(1)
Hg_3_L_2_^6+^ + H^+^ = Hg_3_HL_2_^7+^	10.4(2)
Hg_3_L_2_H^7+^ + H^+^ = Hg_3_L_2_H_2_^8+^	9.0(2)
Hg_2_L^4+^ + HgL^2+^ = Hg_3_L_2_^6+^	2.9(2)
L + 3Hg^2+^ = Hg_3_L^6+^	66.74(5)
Hg_3_L^6+^ + OH^−^ = Hg_3_LOH^5+^	4.83(8)
Hg_2_L^4+^ + Hg^2+^ = Hg_3_L^6+^	18.36(8)
H_7_L^7+^ + HgCl_4_^2−^ = [(H_7_L)HgCl_4_]^5+^	2.7(1)
H_8_L^8+^ + HgCl_4_^2−^ = [(H_8_L)HgCl_4_]^6+^	2.8(1)

**Figure 2:**
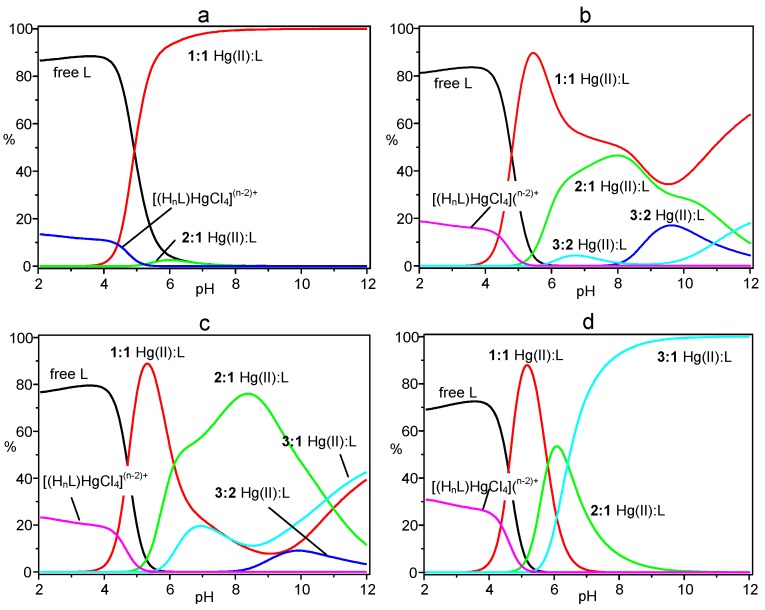
Cumulative distribution diagrams for the system Hg(II)/L. (**a**) [Hg(II)] = [L] = 1 × 10^−3^ M; (**b**) [Hg(II)] = 1.5 × 10^−3^ M, [L] = 1 × 10^−3^ M; (**c**) [Hg(II)] = 2 × 10^−3^ M, [L] = 1 × 10^−3^ M; (**d**) [Hg(II)] = 3 × 10^−3^ M, [L] = 1 × 10^−3^M. [Cl^−^] = 0.1 M in all cases.

The results shown in the table evidence the ability of L to form a variety of complexes with Hg(II), including 1:1, 3:2, 2:1 and 3:1 metal-to-ligand species, of different protonation states, as well as the anion complexes [(H_7_L)HgCl_4_]^5+^ and [(H_8_L)HgCl_4_]^6+^. Cumulative species distribution diagrams calculated [17,18] for the formation of these complexes in 1:1, 3:2, 2:1 and 3:1 metal-to-ligand molar ratios (R), under the experimental conditions adopted for the potentiometric measurements, are shown in Figure 2. In these diagrams, the percentage of the overall concentration of complexes with a particular metal-to-ligand stoichiometry (all protonation states) are represented as a function of pH. Distribution diagrams showing the formation of the individual species are reported in the Supplementary Material (Figures S1–S4). As can be seen in Figure 2, in a solution containing Hg(II) and L in 1:1 molar ratio (R = 1), the mononuclear complexes are the main species above pH 4.5 becoming the only species from pH 8 above (Figure 2a). Increasing R, complexes of higher nuclearity (greater number of metal ions) become of increasing importance (Figure 2b–d), until for R = 3 only trinuclear complexes are formed in alkaline media (Figure 2d). The adducts formed by the anionic HgCl_4_^2−^ species are always present in acidic solutions (pH < 5) and their formation increases with increasing R. It is interesting to note that, even for R > 1, mononuclear complexes are the main species in solution around pH 5. This is due to the very high stability of the HgL^2+^ complex (logK = 28.17) and to the high tendency of this complex to bear protonation forming stable species (Table 1) that strongly compete, in this pH region, with the binding of additional metal ions.

**Figure 3:**
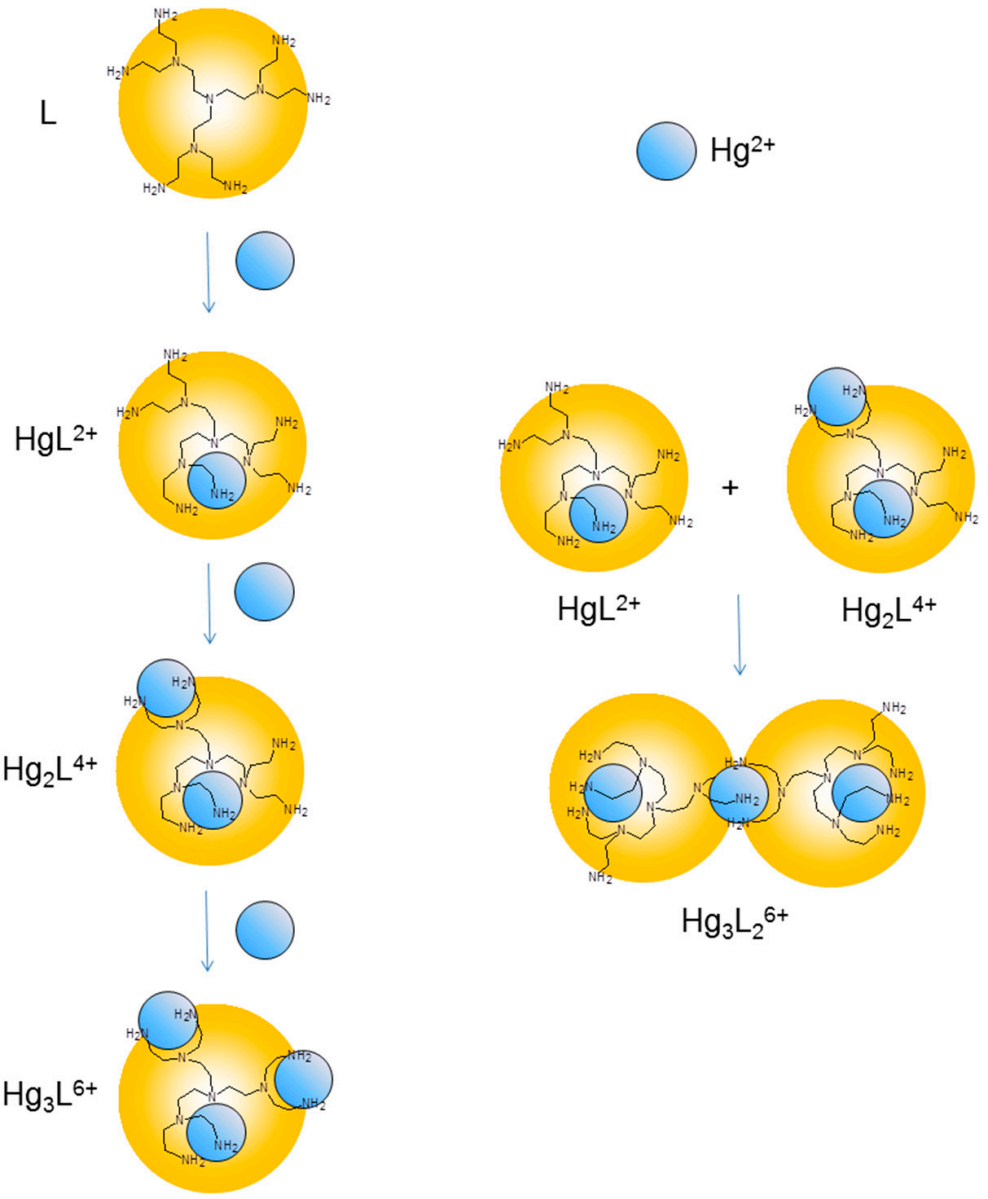
Schematic representation of the coordination environments suggested for the Hg(II) complexes formed by L in solution.

Protonation of primary amine groups in the free ligand L was shown to occur with equilibrium constants log*K* ≥ 8.32, while tertiary ones undergo protonation with log*K* ≤ 5.69 [13]. Table 1 shows that the first three successive protonation constants of HgL^2+^ are greater than the limiting value (log*K* ≥ 8.32) for protonation of primary nitrogens and, accordingly these three protonation stages should involve primary amine groups that are not engaged in metal coordination. The fourth protonation constant (log*K* = 6.13 for HgLH_3_^5+^ + H^+^ = HgLH_4_^6+^, Table 1) is intermediate between the limiting log*K* values for protonation of primary and tertiary amine groups of L. Nevertheless, considering that in the complex, the ligand is not able to expand its structure to minimize the electrostatic repulsion between positive charges as it is able to do it in its metal-free form, even this fourth protonation stage can be ascribed to an uncoordinated primary nitrogen. Successive protonation, the fifth one, has a log*K* value typical of tertiary amine group. All in all, we can reasonably conclude that, in HgL^2+^, the metal ion is coordinated to five ligand donor atoms, while four primary and one tertiary nitrogen atoms keep uncoordinated. By similar reasoning, it is possible to draw some conclusions about the coordinated donor atoms in the different complexes and propose the coordination scheme depicted in Figure 3.

This coordination scheme is consistent with previous results obtained with Ni(II), Zn(II), Cd(II) and Cu(II) and with the crystal structures of the Ni_3_L_2_^6+^ and Cu_3_L^6+^ complexes [13]. It was reported that Ni(II), Zn(II), Cd(II) and Cu(II) form mono- and binuclear complexes with L, while only Cu(II) forms trinuclear species and Ni(II), Zn(II) and Cd(II) form 3:2 metal-to-ligand complexes [13]. Interestingly, Hg(II) forms the whole variety of species and all of them (HgL^2+^, Hg_3_L_2_^6+^, Hg_2_L^4+^, Hg_3_L^6+^) are considerably more stable than the corresponding complexes formed by the other metal ions.

As far as the sequestering ability of L toward Hg(II) is considered, we note that the stability constant of the HgL^2+^ complex (log*K* = 28.17, Table 1) ranks among the highest values shown by the most efficient chelating agents used for Hg(II) sequestration [19,20]. Furthermore, the ligand is able to sustain the coordination of two additional Hg(II) ions, the corresponding equilibrium constants being still very high (log*K* = 20.21 for HgL^2+^ + Hg^2+^ = Hg_2_L^4+^ and log*K* = 18.36 for Hg_2_L^4+^ + Hg^2+^ = Hg_3_L^6+^, Table 1), thus making L an excellent sequestering agent for Hg(II). For instance, calculations performed by means of the computer program HYSS [21] show that upon addition of a millimolar amount of L to an aqueous solution of Hg(II), in a concentration corresponding to the maximum level recorded for the Minamata Bay seawater (3.6 μg/L) and in the presence of Cl^−^ at the common 0.6 M concentration of seawater, the overall concentration of free mercury (Hg(II), HgCl^+^, HgCl_2_, HgCl_3_^−^, HgCl_4_^2−^) is reduced to 1.8 ng/L at pH 7, to 0.06 ng/L at pH 7.5 and to 0.02 ng/l at pH 8, despite the strong competitive effect of chloride. Furthermore, under the same conditions but in the absence of chloride, the concentration of free mercury at these pH values is negligible, becoming appreciable only in very acidic solutions (5 × 10^−5^ ng/L at pH 3). In more alkaline media (pH > 8) the binding ability of L is even greater.

## 3. Experimental Section

### 3.1. General

All starting materials were high purity compounds purchased from commercial sources and used as supplied. Ligand L was synthetized according to a previously described procedure [13].

### 3.2. Potentiometric Measurements

Potentiometric (pH-metric) titrations, used to determine equilibrium constants, were performed in 0.1 M Me_4_NCl aqueous solution at 298.1 ± 0.1 K by using an automated system and a procedure already described [13]. The combined Metrohm 6.0262.100 electrode was calibrated as a hydrogen-ion concentration probe by titration of previously standardized amounts of HCl with CO_2_-free NMe_4_OH solutions and determining the equivalent point by Gran’s method [22], which gives the standard potential, *E*°, and the ionic product of water (p*K*_w_ = 13.83(1) in 0.1 M Me_4_NCl at 298.1 K). A chloride containing medium (0.1 M from the electrolyte) was adopted to take advantage from the competing coordinative effect of chloride in determining the high stability constants of Hg(II) complexes. The computer program HYPERQUAD [16] was used to calculate complex stability constants. Five titrations were performed in the pH range investigated (2.5–11.0) with 1 × 10^−3^ M ligand concentration and Hg^2+^ concentration in the range 0.5[L] ≤ [Hg^2+^] ≤ 2.8[L]. The different titration curves were treated as separated curves without significant variations in the values of the common stability constants. Finally, the sets of data were merged together and treated simultaneously to give the final stability constants. The hydrolysis of metal ion was considered in calculations. Different equilibrium models for the complex systems were generated by eliminating and introducing different species. Only those models for which the HYPERQUAD program furnished a variance of residuals σ^2^ ≤ 9 were accepted. This condition was unambiguously met by a single model. Ligand protonation constants (log*K* = 10.16, 9.98, 9.25, 9.22, 8.57, 8.32, 5.69, 2.60) [13] and stability constants of Hg(II) chloride complexes [19] were taken from the literature.

## 4. Conclusions

The G-2 poly(ethylene imine) dendrimer L is able to bind Hg(II) forming complexes with 1:1, 3:2, 2:1 and 3:1 metal-to-ligand stoichiometries. In acidic solution and in the presence of chloride anions, the protonated species H_7_L^7+^ and H_8_L^8+^ interact with HgCl_4_^2−^ to form the [(H_7_L)HgCl_4_]^5+^ and [(H_8_L)HgCl_4_]^6+^ anion complexes. The stability of the Hg(II) complexes is very high, much higher than the stability previously found for the analogous complexes with Ni(II), Zn(II), Cd(II) and Cu(II), and ranks among the highest values shown by the most efficient chelating agents used for Hg(II) sequestration. High stability is also observed for protonated Hg(II) complexes with L, which makes this polyamine dendrimer an excellent sequestering agent for Hg(II) from acidic to alkaline conditions. Accordingly, L is a promising ligand for the preparation of functionalized activated carbons to be employed in the decontamination of polluted waters containing Hg(II), even in the presence of high chloride concentrations, like in seawater, and in a wide pH range. The preparation of such hybrid material and its application will be the subject of a future study.

## Supplementary Materials

Supplementary materials can be accessed at: http://www.mdpi.com/1420-3049/20/03/3783/s1.
