# Supplementary material for: Inorganic Mercury Sequestration by a Poly(ethylene imine) Dendrimer in Aqueous Solution
Source: Molecules. 2015 Feb 26;20(3):3783–90. doi: 10.3390/molecules20033783 (PMC6272757; doi:10.3390/molecules20033783)
Supplement: Supplementary file 1 [file molecules-20-03783-s001.pdf]

Supplementary Materials

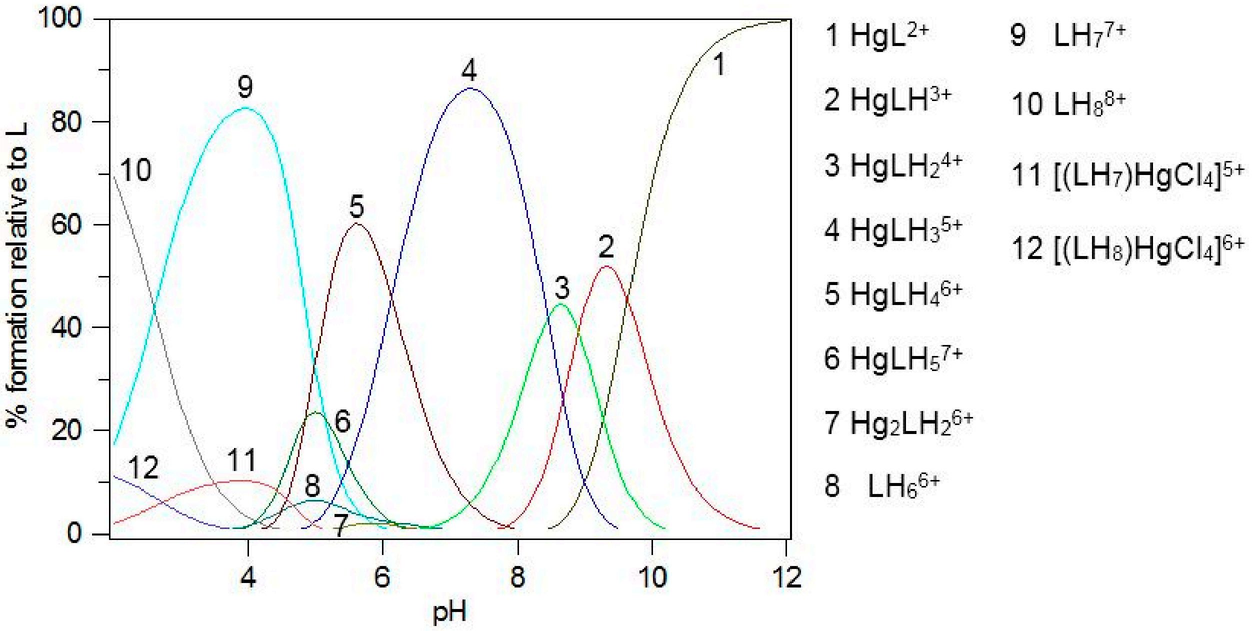

Figure S1. Distribution diagram of the species formed in the system Hg(II)/L in the presence of [Hg(II)] = [L] = 1×10^{-3} M, [Cl^-] = 0.1 M, 298.1 K.

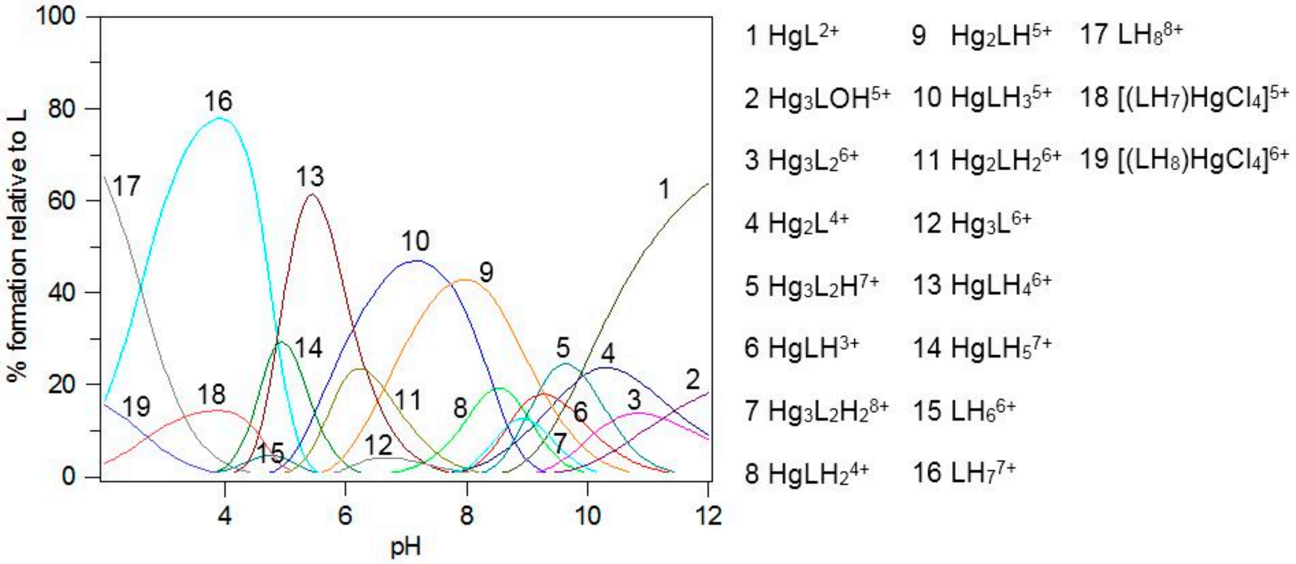

Figure S2. Distribution diagram of the species formed in the system Hg(II)/L in the presence of [Hg(II)] = 1.5×10^{-3} M, [L] = 1×10^{-3} M, [Cl^-] = 0.1 M, 298.1 K.

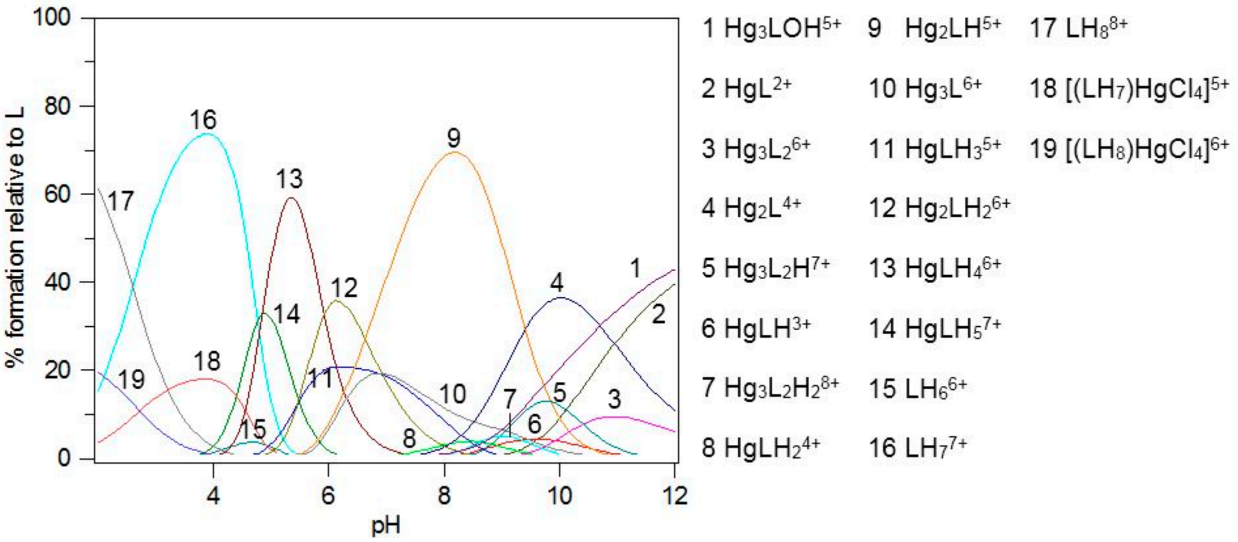

Figure S3. Distribution diagram of the species formed in the system Hg(II)/L in the presence of [Hg(II)] = 2 × 10^{-3} M, [L] = 1 × 10^{-3} M, [Cl^-] = 0.1 M, 298.1 K.

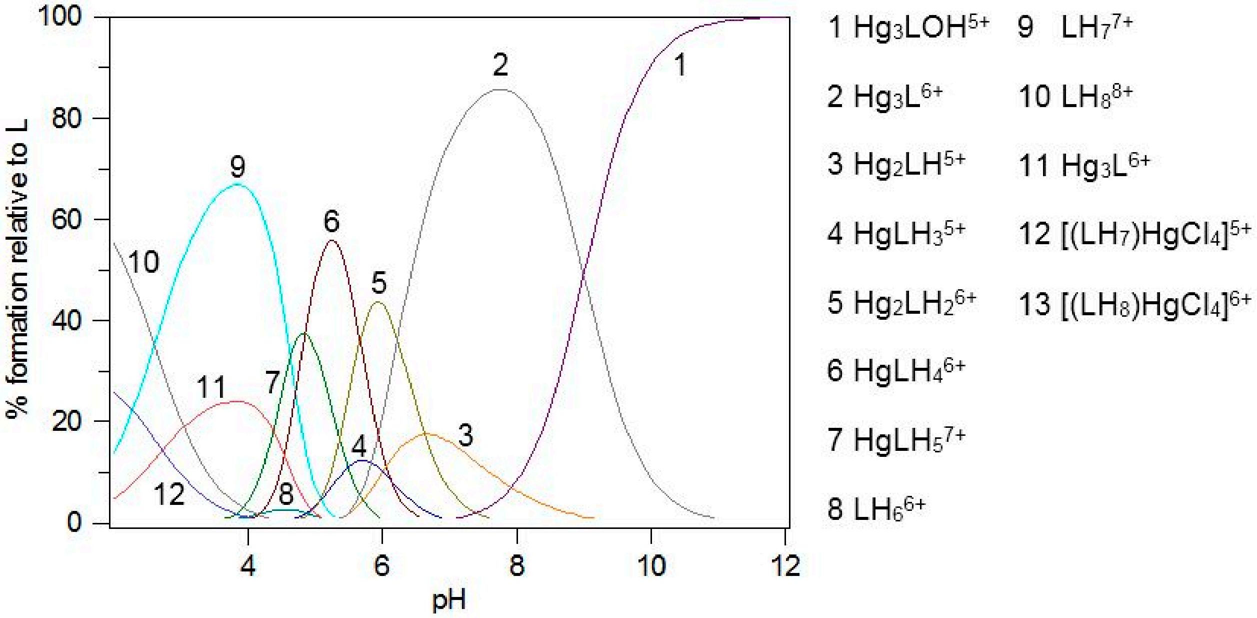

Figure S4. Distribution diagram of the species formed in the system Hg(II)/L in the presence of [Hg(II)] = 3 × 10^{-3} M, [L] = 1 × 10^{-3} M, [Cl^-] = 0.1 M, 298.1 K.
